# Supplementary material for: The Tools for Integrated Management of Childhood Illness (TIMCI) study protocol: a multi-country mixed-method evaluation of pulse oximetry and clinical decision support algorithms
Source: Glob Health Action. 2024 Apr 29;17(1):2326253. doi: 10.1080/16549716.2024.2326253 (PMC11060010; doi:10.1080/16549716.2024.2326253)
Supplement: TIMCI protocol manuscript_Supplement.docx [file ZGHA_A_2326253_SM0912.docx]

**Supplement: Overview of the data collected within TIMCI and their sources of information.**

| TIMCI data activities | Data collection tool | Children sub-sample (where relevant) | Source of data | Collected data |
| --- | --- | --- | --- | --- |
| Pragmatic cluster randomised controlled trial (RCT)  &  Quasi-experimental pre-post study | Facility ODK questionnaire | All screened children | Caregivers at study facilities, prior to consultation | Eligibility |
|  |  | All children enrolled at Day 0 | Caregivers at study facilities, prior to consultation | Contact details and sociodemographic data |
|  |  | All children enrolled at Day 0 or subsequently attending any study facility during their follow-up period | Caregivers at study facilities, prior to consultation | Reported reason for attendance and prior care-seeking |
|  |  | All children enrolled at Day 0 or subsequently attending any study facility during their follow-up period | Caregivers at study facilities, after consultation | Reported clinical management (including referral, treatment and follow-up advice) |
|  |  | All children enrolled at Day 0 or subsequently attending any study facility during their follow-up period | Clinical records from study facilities, after consultation | Recorded consultation data (including assessments performed such as clinical measurements and diagnostic tests, as well as diagnosis(es) and management) |
|  | Day 7 ODK questionnaire | All enrolled children | Caregivers reached by phone (or alternative mechanisms in Tanzania) | Reported health status (including severe complications) and care-seeking since Day 0 |
|  | Day 28 ODK questionnaire | All children enrolled in the pragmatic cluster RCT | Caregivers reached by phone (or alternative mechanisms in Tanzania) | Reported health status (including severe complications) and care-seeking since Day 0 |
|  | Hospital ODK questionnaire | All enrolled children reported to have attended a hospital (or been admitted to a primary care facility) or lost to follow-up at Day 7 | Clinical records from higher-level facilities or inpatient wards of primary care facilities | Recorded clinical management (including basic clinical, admission and outcome data) |
|  | Weekly facility assessment ODK questionnaire | N/A | Non-participant observations + clarifications from relevant facility staff | Weekly update on staffing, pulse oximetry usage and drug stockouts |
| Routine data | medAL-*reader* application with country-specific clinical algorithms (ePOCT+) | All children consulted with the CDSA in equipped intervention facilities | CDSA records (medAL-*data* database) | Recorded individual routine consultation data (including clinical history, examination, tests, diagnosis(es) and management) |
|  | N/A | N/A | National HMIS | Aggregate routine facility data |
| Service provision assessments (modified Demographic and Health Survey) | Facility assessment ODK questionnaire | N/A | Facility managers + other relevant facility staff | Reported basic information on infrastructure, staffing, services, equipment, supplies and documentation with a focus on factors relevant to child health, pulse oximetry and digital health |
|  | Healthcare provider interview ODK questionnaire | N/A | Healthcare providers | Sociodemographics (including qualifications and training), reported experience with the TIMCI intervention and perceptions of facility working conditions |
|  | Sick child observation ODK tool | All children enrolled in SPA | Standardised observations of consultations + clarification from healthcare providers | Observed clinical assessment, diagnosis(es) and managements, including IMCI, pulse oximetry and CDSA adherence |
|  | Structured caregiver exit interview ODK questionnaire | All children enrolled in SPA | Caregivers | Reported experience of care, understanding of management and post-consultation plans |
| Process mapping & time-flow | Process mapping tool | N/A | Non-participant observations + clarifications from facility staff | Observation notes, static and functional facility maps |
|  | Time-flow ODK tool | All children enrolled in time-flow | Individual participant observations | Timestamp data of the start and end of each steps within the patient flow from facility arrival to exit (including waiting, registration and consultation times)s |
| Healthcare providers in-depth interviews | IDI ODK tool for healthcare providers | N/A | Healthcare providers | Sociodemographic data, audio recordings following semi-structured interview guide on acceptability and usability of interventions and experience of care |
| Caregivers in-depth interviews | IDI ODK tool for caregivers | N/A | Caregivers | Sociodemographic data, audio recordings following semi-structured interview guide on acceptability of interventions and experience of care |
| Stakeholder survey + Key informant interviews | KII ODK tool | N/A | Key informants | Sociodemographic data, audio recordings of interviews following semi-structured interview guide on implementation of interventions over time to inform a package at scale |
|  | Online ODK survey | N/A | Stakeholders involved in policy, implementation or research in child health and / or the interventions at international, national, or sub-national level | Reported perspectives on implementation, policy and research |
| Project data review | Document review matrix | N/A | Project data, including PATH project reports, minutes and M&E data | Key comparative characteristics of reviewed documents |
| Economic evaluation | Medical personnel cost ODK questionnaire | N/A | Facility managers + other relevant facility staff | Reported medical staffing and associated costs |
|  | Non-medical personnel cost ODK questionnaire | N/A | Facility managers + other relevant facility staff | Reported non-medical staffing and associated costs |
|  | Hospital costs | N/A | Government hospital | Out of pocket costs for referred patients |
|  | N/A | N/A | Government databases, facility records, supplemented by data from other sub-studies and the literature where necessary | Training for staff involved in delivering the programme, delivery of the intervention (with annualised capital costs) |
|  | Modelled cost-effectiveness | N/A | Secondary |  |
